# Mealtime Assistance by Family and Professional Caregivers: An Observational Study of Cognitively Impaired Older Adults in Hospitals and Nursing Homes

**DOI:** 10.3390/nursrep16010006

**Published:** 2025-12-24

**Authors:** Hui-Chen (Rita) Chang, FungKuen (Tebbin) Koo, Juyang (Amy) Hui, Hansen (Cindy) Tang, Wenpeng You

**Affiliations:** 1 School of Nursing and Midwifery, Parramatta South Campus, Western Sydney University, Parramatta, NSW 2151, Australia; 2 School of Medicine, Parramatta South Campus, Western Sydney University, Parramatta, NSW 2151, Australia; 20554545@student.westernsydney.edu.au

**Keywords:** cognitive impairment, dementia, malnutrition, mealtime assistance, feeding difficulties, family caregivers, nursing homes

## Abstract

**Background:** Malnutrition is common among older adults with cognitive impairment and contributes to frailty and poorer health outcomes. Many individuals with dementia require mealtime assistance, yet differences in caregiving practices across hospital and nursing home settings remain underexplored. **Aim:** The aim of this study was to compare eating encouragement practices, feeding skills, feeding difficulties, and nutritional status between family caregivers in hospitals and professional caregivers in nursing homes. **Methods:** A cross-sectional observational study was conducted between June 2020 and December 2023 in New South Wales, Australia. The study included 82 older adults (≥65 years) with cognitive impairment: 31 hospital patients supported by family caregivers and 51 nursing home residents supported by assistant nurses. Eating encouragement, feeding skills, and feeding difficulties were assessed using structured observation tools, and nutritional status was evaluated using the Mini Nutritional Assessment–Short Form (MNA-SF). Group differences were analysed using chi-square tests and independent t-tests (*p* < 0.05). **Results:** Family caregivers in hospitals demonstrated stronger relational and engagement-based practices, including consistent handwashing (χ^2^ = 31.945, *p* < 0.001), encouraging self-feeding (χ^2^ = 21.678, *p* < 0.001), verbal cueing (χ^2^ = 12.083, *p* = 0.002), touch prompting (χ^2^ = 51.817, *p* < 0.001), and sitting face to face (χ^2^ = 38.697, *p* < 0.001). Nursing home caregivers showed more advanced technical skills, such as task simplification (χ^2^ = 54.135, *p* < 0.001), mirroring (χ^2^ = 78.456, *p* < 0.001), hand-over-hand guidance (χ^2^ = 73.076, *p* < 0.001), mouth- and lip-opening techniques (both χ^2^ = 81.000, *p* < 0.001), and stronger choking management (*p* < 0.001). Feeding difficulties also differed: refusal behaviours were more common in nursing homes, while distraction and oral–motor issues were more frequent in hospitals. Overall, nursing home residents had significantly poorer nutritional status (t = −12.592, *p* < 0.001). **Conclusions:** Family caregivers provide stronger relational support, whereas professional caregivers demonstrate superior technical competence. Integrating these complementary strengths may enhance mealtime care and reduce malnutrition among cognitively impaired older adults.

## 1. Background

Malnutrition is a major public health concern among older adults, contributing to frailty, reduced muscle strength, impaired immunity, higher vulnerability to infections, and increased morbidity and mortality [1,2]. The risk is particularly pronounced among those with cognitive impairment, including dementia, who experience reduced self-feeding ability, behavioural symptoms such as food refusal, and diminished executive function that collectively compromise nutritional intake [3]. In Residential Aged Care Facilities (RACFs), approximately one-third of residents are malnourished [1], and prevalence is even higher in hospitals, where one observational study reported malnutrition rates above 70% among geriatric inpatients [4]. These figures underscore the urgent need to strengthen nutritional care across both acute and long-term care settings.

Hospital environments present unique challenges. Acute wards are characterised by frequent interruptions, time pressures, and competing clinical demands, many of which coincide with mealtimes [5]. As a result, nutritional care is often deprioritised, placing patients—especially those with dementia—at elevated risk of insufficient intake. Although RACFs aim to support residents’ quality of life, staff similarly face time constraints and multiple responsibilities that may limit their capacity to provide consistent mealtime assistance [5]. Understanding these context-specific constraints is essential to improving nutritional care.

Person-centred care (PCC) is widely recognised as fundamental to effective nutritional support, emphasising care that reflects individual histories, preferences, and cultural backgrounds. PCC is associated with improved eating behaviours, greater autonomy, and better nutritional outcomes [6,7,8,9]. However, staff commonly report challenges implementing PCC at mealtimes due to patient variation, limited time, and lack of confidence in adapting feeding strategies [10]. Educational interventions show promise in addressing these gaps. Training programmes focused on mealtime practices and social interaction skills have been shown to reduce malnutrition, enhance staff confidence, and improve nutritional outcomes [11,12,13,14]. For instance, observational feedback in dementia care units has reduced unintentional weight loss to below national averages [12,13], demonstrating the practical benefits of targeted training.

Survey evidence from Australian RACFs reinforces these training needs. One study found staff scored an average of only 4.67 out of 10 on nutritional knowledge, with almost half reporting insufficient understanding of nutritional assessment and many unaware of residents’ specific feeding difficulties [2]. Without adequate preparation, staff may default to task-focused approaches that prioritise efficiency over engagement, unintentionally diminishing both mealtime experience and intake.

While the central role of professional caregivers is well documented, the contribution of family caregivers during mealtimes remains underexplored. Family members frequently participate in hospital care, motivated by responsibility, cultural expectations, and intimate knowledge of the patient’s preferences [15]. Their presence can enhance the mealtime environment by providing emotional support, reducing anxiety, and promoting intake through familiar routines [15]. This aligns with principles of patient- and family-centred care, which have been associated with improved care quality, stronger trust, and greater staff satisfaction [16]. However, little research has examined the feeding assistance practices of family caregivers in hospital settings, representing a significant knowledge gap. Given rising healthcare demands and workforce pressures, family caregivers may be an underutilised resource in supporting mealtime care [15,16,17].

Cultural appropriateness is another essential dimension of nutritional care. Food is deeply tied to cultural identity, shaping acceptance, preferences, and intake [18]. Failure to provide culturally appropriate meals can lead to refusal, reduced intake, and weight loss, increasing risks of frailty, falls, and functional decline [12]. Conversely, culturally tailored meals improve satisfaction, trust, and nutritional outcomes [12]. As such, cultural competency is a critical element of PCC and should inform mealtime assistance across settings.

Taken together, the high prevalence of malnutrition in both hospitals and RACFs, the challenges faced by nursing staff, the under-recognised role of family caregivers, and the importance of culturally sensitive practice highlight the need for more comprehensive research into mealtime assistance for cognitively impaired older adults. Such research can identify contextual strengths and gaps, inform tailored interventions, and guide training programmes that integrate the complementary strengths of family and professional caregivers.

This study aimed to address these gaps by conducting a comparative observational analysis of feeding assistance for cognitively impaired older adults in hospital and nursing home settings. Specifically, the objectives were to

1. compare caregiver behaviours—including eating encouragement and technical feeding skills—between family caregivers in hospitals and nursing staff in nursing homes;
2. compare patient outcomes, including feeding difficulties and nutritional status, across these two settings.

By addressing these aims, the study provides evidence to inform future training, policy development, and culturally responsive care models aimed at improving mealtime experiences and reducing malnutrition in this vulnerable population.

## 2. Methods

### 2.1. Study Design and Setting

This cross-sectional observational study examined mealtime care practices for cognitively impaired older adults across two care environments in New South Wales, Australia: a sub-acute public hospital geriatric ward and a Residential Aged Care Facility (RACF; hereafter referred to as “nursing home”). The aim was to document naturally occurring mealtime behaviours without introducing any intervention. In the hospital setting, mealtime assistance was provided by family caregivers, whereas in the nursing home, all assistance was delivered exclusively by trained Assistants in Nursing (AINs). Ethical approval for the study was granted by the Western Sydney University Human Research Ethics Committee (HREC H15406). Caregivers were aware that observational data collection would occur; however, they were not informed of the specific timing of observations to minimise the Hawthorne effect.

### 2.2. Participants

#### 2.2.1. Older Adults

Patients and residents were eligible if they

- were aged ≥ 65 years;
- consumed oral meals (non-artificial feeding);
- had cognitive impairment confirmed by a formal dementia diagnosis, MoCA score, or observed behavioural indicators.

Individuals receiving enteral or parenteral nutrition, or those who were comatose, were excluded. These differences in eligibility characteristics reflect the typical care populations served by each site and were not the result of selective recruitment.

#### 2.2.2. Caregivers

Caregivers were the primary individuals providing mealtime assistance: family caregivers in the hospital and AINs in the nursing home.

During screening, 42 hospital patients were eligible, but 31 were assisted by family members and 11 by staff. To maintain two clearly defined comparison groups, staff-assisted hospital patients were excluded, yielding a final sample of 31 hospital participants and 51 nursing home participants.

### 2.3. Sampling and Recruitment

Purposive sampling was used to recruit participants between June 2020 and December 2023. Recruitment was coordinated by the hospital’s Clinical Nurse Educator (CNE) and the nursing home’s Nursing Unit Manager (NUM), who screened for eligibility using a standard protocol. Written informed consent was obtained from participants or their Substitute Decision-Makers and from caregivers.

Sample size was calculated using G*Power (3.1.9.7) (two-tailed independent *t*-test; α = 0.05; power = 0.95; effect size d = 0.80), requiring a minimum of 70 participants. The final sample of 82 exceeded this threshold.

## 3. Measures

### 3.1. Demographic and Mealtime Characteristics

A structured data form was used to capture demographic and clinical information for older adult participants, including age, gender, cognitive status, the level of mealtime assistance required, and the proportion of the meal consumed. Caregiver demographic data were collected only for nursing home staff, as such information was not routinely recorded for family caregivers in the hospital setting.

The level of mealtime assistance was assessed by the trained research assistant during direct observation and categorised using a standardised protocol as: (1) self-feeding (no support required), (2) partial assistance (support needed for selected tasks such as utensil use or pacing), or (3) total assistance (full caregiver feeding). Meal-consumption categories (25%, 50%, 75%, 100%) were converted to proportional values (0.25–1.00) for quantitative analysis.

### 3.2. Eating Encouragement (11 Items)

Relational mealtime behaviours were assessed using the Eating Encouragement Checklist. Ten positive behaviours, such as verbal cueing, reassurance and sitting face to face, were scored as Yes or No. One negative behaviour (infantilisation) resulted in a one-point deduction. Higher total scores indicated stronger relational engagement.

### 3.3. Feeding Skills (12 Items)

Technical mealtime practices were assessed using the 12-item Feeding Skills Checklist, which evaluated behaviours such as multisensory cueing, task simplification, mouth- and lip-opening techniques, swallowing support, and responses to choking risk. Items were scored as Yes/No, with higher scores indicating more advanced use of technical feeding strategies.

Both the Eating Encouragement and Feeding Skills checklists were developed from existing dementia mealtime literature and further refined through a structured pilot-testing process to ensure clarity and consistent application. Pilot testing was conducted across several mealtime observations, during which each item was reviewed for behavioural specificity, clarity of operational definitions, and feasibility in real-time observation. Ambiguous items were reworded, redundant behaviours were removed, and scoring guidance was clarified based on feedback from the research assistant and supervisory team. The final 11-item and 12-item checklists therefore represent a combination of published dementia-mealtime observational tools and field-tested refinements generated during the pilot phase.

### 3.4. Feeding Difficulties

The Feeding Difficulty Index (FDI) was used to observe resident feeding challenges across four domains: distractibility, food-procurement difficulty, refusal behaviours, and motor impairment. Each of the 19 items was rated from 0 (not observed) to 3 (>6 occurrences), generating a total score from 0 to 72, with higher scores indicating more severe feeding difficulties.

### 3.5. Nutritional Status

Nutritional status was assessed using the Mini Nutritional Assessment–Short Form (MNA-SF), which includes six items assessing food intake, recent weight loss, mobility, psychological stress/acute illness, neuropsychological problems, and BMI or calf circumference. Total scores range from 0 to 14 and classify participants as well-nourished (12–14), at risk of malnutrition (8–11), or malnourished (0–7).

The research assistant received structured training in the use of the observational checklists, including calibration exercises and supervised pilot sessions to support consistent application.

### 3.6. Data Collection

Data were collected during routine mealtimes. The research assistant observed one mealtime episode per participant, positioning themselves discreetly to minimise observer effects. For each participant, the MNA-SF and FDI assessments were completed, and the relevant feeding-skill checklist was completed for the assisting caregiver. Additional contextual information, such as interruptions, environmental disturbances, and atypical behaviours was documented. Clinical and demographic information was obtained from medical records and through consultation with clinical staff.

The research assistant completed structured training prior to data collection. This training included familiarisation with all checklist items, review of exemplar videos drawn from dementia mealtime literature, and supervised practice sessions in both care settings. Pilot observations were conducted to refine item definitions, scoring procedures, and behavioural indicators in collaboration with the research team. The research assistant commenced formal data collection only after demonstrating scoring concordance with the lead investigator.

### 3.7. Data Analysis

Data were analysed using IBM SPSS Statistics (Version 29). Missing data were assessed across all observational and nutritional variables, and no missing values were identified; therefore, no imputation procedures were required. Analyses were restricted to bivariate comparisons, consistent with the exploratory design and sample size limitations, and no causal inferences were drawn.

Group differences in continuous outcomes (Eating Encouragement Score, Feeding Skills Score, Feeding Difficulty Index Score, and MNA Score) were examined using independent-sample t-tests. Assumptions for parametric testing were reviewed prior to analysis. Normality was assessed using Shapiro–Wilk tests and visual inspection of histograms and Q–Q plots. For the primary outcome (Eat_score), the hospital group showed evidence of non-normality (W = 0.865, *p* = 0.001), while the nursing home group approximated normality (W = 0.960, *p* = 0.086). Deviations were mild on visual inspection, and t-tests are robust to moderate non-normality with sample sizes of this magnitude; therefore, analyses proceeded using parametric tests. Homogeneity of variance was evaluated using Levene’s test, with results confirming the suitability of the equal-variance or unequal-variance estimates reported.

Given the number of behavioural items examined, chi-square tests were used to compare categorical feeding behaviours between settings. Effect sizes (Cohen’s d with Hedges’ correction) were calculated for all t-tests and reported to support interpretation of the magnitude of differences between groups. Because multiple comparisons increase the risk of Type I error, findings are interpreted cautiously, and emphasis is placed on the consistency and clinical relevance of patterns rather than on individual *p*-values.

All analyses were exploratory and descriptive, intended to characterise observed differences in mealtime behaviours and nutritional outcomes. Multivariable modelling was not conducted due to insufficient sample size to adjust reliably for multiple covariates; this constraint is acknowledged in the Section 5.7.

## 4. Results

### 4.1. Participant Characteristics (Table 1)

Eighty-two older adults were included: 31 hospital patients assisted by family caregivers and 51 nursing home residents assisted by AINs. The groups were similar in age (hospital: mean 88.35, SD 5.30; nursing home: mean 87.80, SD 6.32) and mealtime duration (hospital: 23.9 min, SD 8.44; nursing home: 23.25 min, SD 5.52).

**Table 1:** Demographic and clinical characteristics of older adult participants and caregiver profiles across hospital and nursing home settings.

Characteristic	Hospital (*n* = 31)	Nursing Home (*n* = 51)
Gender, *n* (%)	Female: 12 (38.7) Male: 19 (61.3)	Female: 35 (68.6) Male: 16 (31.4)
Age, mean (SD)	88.35 (5.30)	87.8 (6.32)
Duration of stay	6.77 days (4.49)	3.54 years (2.71)
Ethnicity, *n* (%)	English speaking: 22 (71.0) non-English background: 9 (29.0)	Chinese speaking: 50 (98.0) non-Chinese background: 1 (2.0)
Education level, *n* (%)	Primary: 12 (38.7) High school: 10 (32.3) Higher education: 8 (25.8) Not stated: 1 (3.2)	Primary: 12 (23.5) High school: 18 (35.3) Higher education: 19 (37.3) Not stated: 2 (3.9)
Cognition	Dementia diagnosis: 23 (74.2) No formal diagnosis: 8 (25.8)	MoCA score: 14.39 (8.73)
Antipsychotic medication, *n* (%)	Yes: 10 (32.3) No: 21 (67.7)	Yes: 16 (31.4) No: 35 (68.6)
Level of assistance, *n* (%)	Partial: 19 (61.3) Total: 12 (38.7)	Self-feeding: 18 (35.3) Partial: 18 (35.3) Total: 15 (29.4)
Feeding time, mean (SD)	23.9 (8.44) min	23.25 (5.52) min
Portion of meal consumed *n* (%)	25%: 13 (41.9) 50%: 5 (16.1) 75%: 6 (19.4) 100%: 7 (22.6) Overall mean (SD): 0.56 (0.31)	25%: 1 (1.9) 50%: 6 (11.8) 75%: 34 (66.7) 100%: 10 (19.6) Overall mean (SD): 0.76 (0.16)
Caregiver characteristics	–	Female AINs: 51 (100%) Education: Diploma (100%) Mean years of experience: 3.76 (2.57)

Notes: Results are rounded to one or two decimal places.

Gender profiles differed: the hospital cohort was predominantly male (61.3%), whereas the nursing home cohort was predominantly female (68.6%). Cognitive status was also setting specific. Most hospital patients had a formal dementia diagnosis (*n* = 23; 74.2%), while cognition in the nursing home was assessed using the MoCA, with a mean score of 14.39 (SD 8.73), indicating moderate-to-severe impairment.

Ethnicity and language reflected the demographic characteristics of each site. The hospital primarily served English-speaking patients (71.0%), whereas the nursing home predominantly cared for older adults of Chinese cultural background (98.0% Chinese speaking).

Marked differences were observed in meal consumption. Hospital patients consumed a smaller proportion of their meals on average (mean 0.56, SD 0.31) compared with nursing home residents (mean 0.76, SD 0.16). Notably, 41.9% of hospital patients consumed only 25% of their meal, compared with just 1.9% of nursing home residents. This substantial disparity likely reflects contextual and organisational differences: hospital mealtimes were frequently interrupted by clinical procedures and lacked consistent, trained feeding support, whereas the nursing home provided structured dining routines with continuous assistance from trained AINs.

All mealtime assistance in the nursing home was provided by female AINs, all of whom held diploma-level qualifications and had an average of 3.76 years (SD 2.57) of experience. Mealtime environments also differed substantially between settings. In the hospital, participants ate individually in their rooms using standard overbed tables. In contrast, nursing home residents typically consumed meals in a communal dining room or shared lounge areas, consistent with usual facility routines.

### 4.2. Eating Encouragement Practices (Table 2)

Significant differences in eating encouragement behaviours were observed between settings. Hand-washing before feeding showed a large difference (χ^2^ = 31.945, df = 3, *p* < 0.001), with hospital caregivers demonstrating more consistent hygiene. All caregivers in both settings avoided hurrying residents, with no variability on this item.

**Table 2:** Eating Encouragement: Chi-Square and t-Test Results.

Eating Encouragement Item	Test Result	*p*-Value	Interpretation
1. Washed hands before feeding	χ^2^ = 31.945 (df = 3)	<0.001	Large difference; hospitals showed more consistent hand-washing practice.
2. Did not hurry the resident eating	–	–	No variation; all caregivers avoided hurrying.
3. Fed one resident at a time	χ^2^ = 37.621 (df = 3)	<0.001	Nursing homes much less likely to feed one resident at a time.
4. Did not interrupt feeding	χ^2^ = 10.550 (df = 3)	0.014	Modest but significant difference in avoiding interruptions.
5. Encouraged self-feeding	χ^2^ = 21.678 (df = 3)	<0.001	Hospital caregivers encouraged self-feeding more often.
6. Sat and faced the resident	χ^2^ = 38.697 (df = 3)	<0.001	Very large difference; hospitals more likely to face the resident.
7. Used verbal cueing	χ^2^ = 12.083 (df = 2)	0.002	Verbal cueing more common in hospitals.
8. Used touch to encourage eating	χ^2^ = 51.817 (df = 3)	<0.001	Strong difference; touch encouragement common in hospitals.
9. Talked to the resident while feeding	χ^2^ = 14.018 (df = 2)	<0.001	Conversation during feeding more common in hospitals.
10. Used reinforcement (e.g., reward)	χ^2^ = 82.000 (df = 3)	<0.001	Very large difference; reinforcement rarely used in nursing homes.
11. Used negative enhancement (e.g., infantilisation)	χ^2^ = 52.000 (df = 2)	<0.001	Rare overall but more frequent in nursing homes.
Total Eating Encouragement Score (Eat_score)	t = 3.183 (df = 80) Mean difference = 1.08295% CI (mean difference): 0.406 to 1.759 Effect size (Cohen’s d): 1.4995% CI (d): 0.26 to 1.18	0.002	Hospitals scored significantly higher on overall encouragement (large effect size).

Feeding one resident at a time differed markedly (χ^2^ = 37.621, df = 3, *p* < 0.001), with nursing home staff significantly less likely to provide one-to-one feeding support. Avoiding interruptions during feeding also varied (χ^2^ = 10.550, df = 3, *p* = 0.014), indicating a modest but meaningful gap in uninterrupted mealtime care.

Hospital caregivers more frequently encouraged self-feeding (χ^2^ = 21.678, df = 3, *p* < 0.001) and were much more likely to sit and face the resident (χ^2^ = 38.697, df = 3, *p* < 0.001), reflecting stronger person-centred engagement. Verbal cueing (χ^2^ = 12.083, df = 2, *p* = 0.002), touch-based encouragement (χ^2^ = 51.817, df = 3, *p* < 0.001), and conversational interaction during feeding (χ^2^ = 14.018, df = 2, *p* < 0.001) were also significantly more common in hospitals.

Reinforcement strategies (e.g., rewards) showed one of the largest differences (χ^2^ = 82.000, df = 3, *p* < 0.001), being rarely used in nursing homes. Negative enhancement (e.g., infantilisation) was uncommon overall but recorded more frequently in nursing homes (χ^2^ = 52.000, df = 2, *p* < 0.001).

The total Eating Encouragement score confirmed these patterns: hospital caregivers scored significantly higher than nursing home caregivers (*t*(80) = 3.183, *p* = 0.002), with a large effect size (d ≈ 1.49), indicating substantially stronger overall encouragement practices in hospital settings.

### 4.3. Feeding Skills Performance (Table 3)

Feeding skills profiles differed significantly across settings. Multisensory cueing was more frequently used in hospitals (χ^2^ = 21.449, df = 2, *p* < 0.001). In contrast, nursing homes relied more heavily on task simplification and sequencing (χ^2^ = 54.135, df = 3, *p* < 0.001).

**Table 3:** Feeding Skills by Care Setting (Hospital vs. Nursing Home): Chi-Square and *t*-Test Results.

Feeding Skills Item	Test Result	*p*-Value	Interpretation
1. Multisensory cueing	χ^2^ = 21.449 (df = 2)	<0.001	Hospitals used multisensory cueing more frequently.
2. Task simplification and sequencing	χ^2^ = 54.135 (df = 3)	<0.001	Nursing homes relied more on step-by-step task simplification.
3. Mirroring	χ^2^ = 78.456 (df = 3)	<0.001	Mirroring techniques were far more common in nursing homes.
4. Hand-over-hand approach	χ^2^ = 73.076 (df = 3)	<0.001	Nursing homes used hand-over-hand guidance substantially more often.
5. Chaining and end-chaining	χ^2^ = 82.000 (df = 3)	<0.001	Chaining techniques were rarely used in hospitals.
6. Bridging (symbolic object use)	χ^2^ = 82.000 (df = 3)	<0.001	Bridging was almost exclusively used in nursing homes.
7. Mouth-open techniques	χ^2^ = 81.000 (df = 3)	<0.001	Nursing homes used mouth-open techniques much more frequently.
8. Lip-open techniques	χ^2^ = 81.000 (df = 3)	<0.001	Lip-opening techniques were also more common in nursing homes.
9. Swallowing-assistance techniques	χ^2^ = 78.172 (df = 3)	<0.001	Nursing homes more frequently supported swallowing.
10. Oral stimulation (e.g., water before eating)	χ^2^ = 9.067 (df = 2)	0.011	Oral-stimulation techniques were used slightly more often in nursing homes.
11. Observing choking signs	χ^2^ = 34.425 (df = 3)	<0.001	Nursing home staff more consistently recognised choking signs.
12. Dealing with choking correctly	χ^2^ = 29.129 (df = 2)	<0.001	Nursing homes demonstrated better choking response skills.
Total Feeding Skills Score (FeedSkill_Score)	t = −1.870 (df = 80) (equal variances assumed) Mean difference = −0.64395% CI (mean difference): −1.327 to 0.041 Effect size (Cohen’s d): −0.42695% CI (d): −0.876 to 0.027	0.065 (two-tailed) 0.033 (one-tailed)	Nursing homes had higher overall feeding-skills scores; difference small and only significant with the one-tailed test.

Mirroring techniques (χ^2^ = 78.456, df = 3, *p* < 0.001) and the hand-over-hand approach (χ^2^ = 73.076, df = 3, *p* < 0.001) were substantially more common in nursing homes, indicating greater use of guided and imitative strategies. Chaining and end-chaining (χ^2^ = 82.000, df = 3, *p* < 0.001) and bridging with symbolic objects (χ^2^ = 82.000, df = 3, *p* < 0.001) were almost exclusively observed in nursing homes, suggesting these advanced dementia support techniques were rarely used in hospitals.

Nursing homes also more frequently employed mouth-open and lip-open techniques (both χ^2^ = 81.000, df = 3, *p* < 0.001) and swallowing-assistance strategies (χ^2^ = 78.172, df = 3, *p* < 0.001). Oral stimulation (e.g., water before eating) was used slightly more often in nursing homes (χ^2^ = 9.067, df = 2, *p* = 0.011).

Emergency-related skills were stronger in nursing homes: staff more consistently recognised choking signs (χ^2^ = 34.425, df = 3, *p* < 0.001) and more often responded correctly (χ^2^ = 29.129, df = 2, *p* < 0.001).

The total Feeding Skills score was higher in nursing homes (*t*(80) = −1.870, *p* = 0.065 two-tailed; *p* = 0.033 one-tailed), although the mean difference was small (−0.64). Overall, hospitals emphasised cueing-based encouragement, whereas nursing homes demonstrated more advanced technical and safety-related feeding skills.

### 4.4. Feeding Difficulties (Table 4)

Feeding difficulty patterns differed across settings. Nursing home residents more often displayed refusal-related behaviours, including pushing or resisting food offered by hand (χ^2^ = 7.600, df = 2, *p* = 0.022) and not opening the mouth or biting utensils (χ^2^ = 8.808, df = 3, *p* = 0.032). Delays of ≥1 min before initiating eating were also more frequent in nursing homes (χ^2^ = 9.699, df = 3, *p* = 0.021).

**Table 4:** Feeding Difficulties by Care Setting (Hospital vs. Nursing Home): Chi-Square and *t*-test Results.

Feeding Difficulty Index Item	Test Result	*p*-Value	Interpretation
1. Pushes or resists food offered by hand	χ^2^ = 7.600 (df = 2)	0.022	Resistance to hand-fed food was more frequent in nursing homes.
2. Negative behaviour toward feeder (pushes, hits, kicks, throws objects)	χ^2^ = 0.615 (df = 1)	0.433	No significant difference in overt negative behaviours toward feeders.
3. Inappropriate verbal statements toward feeder	χ^2^ = 1.230 (df = 1)	0.267	Negative verbal responses were uncommon and did not differ significantly between settings.
4. Turns head away or tilts head backward	χ^2^ = 2.256 (df = 2)	0.324	Head-turning or avoidance was similar across settings.
5. Spits out the food	χ^2^ = 1.666 (df = 2)	0.435	Spitting behaviours were infrequent and showed no significant difference.
6. Does not open the mouth or bites the utensils when food is offered	χ^2^ = 8.808 (df = 3)	0.032	Residents in nursing homes were more likely to refuse opening the mouth or bite utensils when food was offered.
7. Leaves the table	χ^2^ = 1.230 (df = 1)	0.267	No significant difference in leaving the table during meals.
8. Cannot sit still (slipping or twisting body)	χ^2^ = 5.664 (df = 2)	0.059	Restlessness during meals was slightly more common in hospitals, but the difference did not reach statistical significance.
9. Does not start to eat for ≥1 min when invited	χ^2^ = 9.699 (df = 3)	0.021	Delayed initiation of eating was more common in nursing homes, with more residents showing longer delays.
10. Becomes drowsy or falls asleep	χ^2^ = 3.090 (df = 3)	0.378	No significant difference in drowsiness or falling asleep during meals.
11. Discontinues eating for over 1 min	χ^2^ = 8.044 (df = 3)	0.045	Pauses in eating for more than one minute were slightly more frequent in nursing homes.
12. Distracted from eating by talking, looking around, or watching TV	χ^2^ = 31.592 (df = 3)	<0.001	Distraction was common in both groups but marked distraction was more frequent among hospital residents.
13. Plays with food (does something with food but does not eat it)	χ^2^ = 1.116 (df = 1)	0.291	Playing with food was rare and did not differ significantly between settings.
14. Unable to successfully pick up food with utensil	χ^2^ = 4.974 (df = 3)	0.174	No statistically significant difference in difficulty picking up food with utensils.
15. Once food is on utensil, unable to get it effectively into the mouth	χ^2^ = 7.663 (df = 3)	0.054	A trend towards more difficulty in hospitals, but this did not reach conventional significance.
16. Uses hand to feed self	χ^2^ = 16.145 (df = 3)	0.001	Hand feeding was substantially more frequent in hospitals.
17. Once food is in the mouth, food dribbles out	χ^2^ = 20.483 (df = 3)	<0.001	Oral leakage of food was observed more often among hospital residents.
18. Continuously chews or holds food without swallowing	χ^2^ = 2.169 (df = 3)	0.538	Continuous chewing or food-holding behaviours were similar across settings.
19. Chokes or gags on food	χ^2^ = 2.212 (df = 2)	0.331	Choking or gagging events were infrequent and did not differ significantly.
Total Feeding Difficulty Score (FDI_Score)	t = 2.08 (df = 72.9; unequal variances) Mean difference = 1.68 (Hospital > Nursing home) 95% CI (mean difference): 0.07 to 3.30 Effect size (Cohen’s d): 0.4095% CI (d): −0.06 to 0.85	0.041 (two-tailed) 0.021 (one-tailed)	Hospital residents had higher overall feeding difficulty scores than nursing home residents, with a small-to-moderate effect size.

In contrast, certain difficulties were more prevalent in hospitals. Hospital patients were more often distracted during meals by talking, looking around, or watching TV (χ^2^ = 31.592, df = 3, *p* < 0.001). They also relied more on hand feeding (χ^2^ = 16.145, df = 3, *p* = 0.001), and food dribbling from the mouth was more frequently observed (χ^2^ = 20.483, df = 3, *p* < 0.001), suggesting greater oral–motor control problems.

Most other feeding difficulty items, including negative behaviours toward the feeder, inappropriate verbal statements, head turning, spitting, leaving the table, drowsiness, difficulties using utensils, prolonged chewing without swallowing, and episodes of choking, did not differ significantly between the groups. Only two items showed trends: difficulty sitting still (χ^2^ = 5.664, df = 2, *p* = 0.059) and failure to transfer food from utensil to mouth (χ^2^ = 7.663, df = 3, *p* = 0.054).

The total FDI score was significantly higher among hospital patients than nursing home residents (*t*(72.9) = 2.077, *p* = 0.041), with a small to moderate effect size (d ≈ 0.40), indicating greater overall feeding difficulties in the hospital group.

### 4.5. Nutritional Status (MNA, Table 5)

Marked differences in nutritional status were observed between settings. A decline in food intake over the previous three months was far more frequent in nursing homes (χ^2^ = 54.852, df = 2, *p* < 0.001). Recent weight loss patterns also differed significantly (χ^2^ = 43.559, df = 3, *p* < 0.001), with nursing home residents showing higher rates of moderate to severe weight loss.

**Table 5:** Mini Nutritional Assessment (MNA) by Care Setting (Hospital vs. Nursing Home): Chi-Square and *t*-Test Results.

MNA Item	Test Result	*p*-Value	Interpretation
A. Decline in food intake (past 3 months)	χ^2^ = 54.852 (df = 2)	<0.001	Food intake decline was far more frequent among nursing home residents.
B. Weight loss (past 3 months)	χ^2^ = 43.559 (df = 3)	<0.001	Weight loss patterns differed significantly, with greater weight loss reported in nursing homes.
C. Mobility	χ^2^ = 42.087 (df = 2)	<0.001	Mobility limitations were substantially more common among nursing home residents.
D. Psychological stress or acute disease	χ^2^ = 45.207 (df = 1)	<0.001	Recent psychological stress or acute illness was more frequently reported in nursing homes.
E. Neuropsychological problems	χ^2^ = 10.098 (df = 2)	0.006	Neuropsychological issues were more common among nursing home residents.
F1. Body Mass Index (BMI)	χ^2^ = 4.233 (df = 3)	0.237	BMI categories did not differ significantly between settings.
Overall MNA Category (malnourished/at risk/normal)	χ^2^ = 40.982 (df = 1)	<0.001	Malnutrition or risk of malnutrition was markedly more common among nursing home residents.
Total MNA Score (MNA_Score)	t = −12.592 (df = 76.78) Mean difference = −4.610 (NH > Hospital) 95% CI: −5.339 to −3.881 Cohen’s d = −2.668 (95% CI: −3.271 to −2.056)	<0.001	Nursing home residents had significantly poorer nutritional status overall (very large effect size).

Mobility was substantially lower in nursing homes (χ^2^ = 42.087, df = 2, *p* < 0.001), and recent psychological stress or acute illness was more common (χ^2^ = 45.207, df = 1, *p* < 0.001). Neuropsychological problems were also more frequently reported among nursing home residents (χ^2^ = 10.098, df = 2, *p* = 0.006).

BMI categories did not differ significantly between groups (χ^2^ = 4.233, df = 3, *p* = 0.237), indicating similar weight-for-height distributions despite differences in other nutritional risk markers.

Overall MNA categories revealed a pronounced disparity: malnutrition or risk of malnutrition was markedly more common in nursing homes (χ^2^ = 40.982, df = 1, *p* < 0.001). Consistently, the total MNA score was significantly lower in nursing home residents (*t*(76.78) = −12.592, *p* < 0.001), with a very large effect size (d ≈ −2.67), indicating substantially poorer nutritional status compared with hospital patients.

## 5. Discussion

This study contributes new comparative evidence on mealtime care for cognitively impaired older adults by contrasting family-provided relational care in hospitals with professionally delivered technical care in nursing homes. These differences mirror patterns described in previous dementia feeding literature, where relational engagement and emotional connection are typically stronger in family caregiving [12,15,17,18], while technical feeding skills and safety practices are more developed among trained care staff [11,13,14,19,20]. Together, the results reinforce the need for integrated relational-technical models consistent with person-centred care (PCC) frameworks.

### 5.1. Relational Care in Hospitals

Family caregivers demonstrated significantly stronger encouragement-based behaviours, including verbal cueing, reassurance, touch, and sustained conversation. These findings are consistent with earlier research showing that family involvement during mealtimes can enhance comfort, reduce agitation, and improve cooperation among older adults with cognitive impairment [15,17]. These relational behaviours align with PCC principles, which emphasise familiarity, identity, and biographical knowledge as key to promoting dignity and engagement [9,10]. Cultural familiarity, which has been emphasised in earlier studies as a key strength of family involvement [12,18], may also have influenced how families supported mealtime interactions.

However, consistent with hospital-based nutrition research, relational strengths alone were not sufficient to produce higher intake. Hospital patients consumed significantly smaller portions and demonstrated greater feeding difficulties, aligning with studies showing that acute illness, environmental disruptions, bedside eating, and frequent clinical interruptions collectively reduce meal intake among older inpatients and contribute to malnutrition [21,22,23]. Previous findings also indicate that family caregivers often lack training in safe feeding, positioning, and choking management [11,13], which corresponds with the low technical skill use observed in hospitals.

### 5.2. Technical Care in Nursing Homes

Nursing home staff demonstrated far stronger technical feeding skills, including task simplification, mirroring techniques, guided hand-over-hand movements, chaining, bridging, and advanced mouth- or lip-opening strategies. These results are consistent with prior work showing that trained staff in dementia care settings employ more structured compensatory strategies and demonstrate better recognition of aspiration and choking risks [20,24,25,26]. Earlier intervention studies also report that technical skill proficiency is linked to improved safety, reduced feeding time, and greater meal completion in RACF populations [11,13,14].

At the same time, relational engagement was less frequent among nursing home staff, reflecting the task-oriented routines, time pressures, and staffing constraints documented extensively in aged-care research [5,10]. This imbalance aligns with studies showing that RACF staff prioritise efficiency and safety over engagement, particularly in high-dementia units where workload is high. The lack of relational strategies in this setting reinforces calls in the literature for combined training that includes both technical skills and relational, communication-focused PCC [9,18].

### 5.3. Feeding Difficulties and Setting-Specific Patterns

The distinct feeding difficulty patterns observed in each setting align with prior studies demonstrating that behaviour profiles differ depending on environmental stability and caregiver support. Nursing home residents displayed more refusal behaviours (e.g., pushing food away, not opening the mouth), consistent with literature describing refusal as common among long-term RACF residents with advanced cognitive impairment [27]. In contrast, hospital patients showed more distraction and oral–motor issues, echoing earlier findings that acute inpatient environments heighten distraction and reduce feeding efficiency [21,22,23,28].

Overall FDI scores were higher in hospitals, reinforcing previous evidence that acute illness severity and environmental instability contribute more strongly to feeding difficulty than cognitive impairment alone.

### 5.4. Nutritional Status (MNA) and Chronic vs. Acute Patterns

A key finding was that nursing home residents had significantly poorer overall MNA scores and were more likely to be malnourished or at risk, despite consuming more food during the observed mealtime. This aligns with prior MNA research showing that RACF residents often experience chronic, cumulative nutritional decline, reflected in long-term issues such as weight loss, reduced habitual intake, mobility limitations, and higher psychological burden [1,2,3,4]. These structural vulnerabilities may overshadow short-term meal intake.

Conversely, hospital patients ate less during the observed meal but had better MNA-SF scores, consistent with earlier reports that short hospital stays are too brief to reflect chronic malnutrition patterns [4]. This underscores that the MNA primarily captures longer-term nutritional risk rather than momentary intake, a distinction supported by previous validation studies.

### 5.5. Implications for Integrated Mealtime Care

The findings reinforce prior research showing that neither relational engagement nor technical feeding skills alone can adequately address malnutrition among older adults with cognitive impairment [9,10,11,13,14]. Consistent with earlier studies, family caregivers demonstrated strong relational behaviours that can enhance comfort, cultural alignment, and willingness to eat, while professional carers provided the technical skills required to support dysphagia, oral–motor impairment, and safety risks.

Evidence from feeding education interventions indicates that integrated training that combines person-centred care principles, multisensory cueing, and safe feeding techniques improves nutritional outcomes and increases caregiver confidence [11,13].

The current results point to clear opportunities for cross-training across settings:

- Family caregivers in hospitals would benefit from targeted guidance on positioning, pacing, and safe swallowing support to complement their relational strengths.
- Professional carers in nursing homes would benefit from enhanced training in relational communication, cultural responsiveness, and engagement strategies that promote autonomy and dignity.

### 5.6. Toward a Combined Relational–Technical Model

Consistent with PCC frameworks and prior research advocating shared caregiving models [15,16,18], these results support the development of collaborative mealtime approaches that draw on the complementary strengths of families and staff. Integrating relational and technical skills has the potential to address not only acute feeding performance but also long-term nutritional risk, thereby reducing malnutrition, enhancing quality of life, and improving health outcomes for cognitively impaired older adults.

### 5.7. Strengths and Limitations

This study has several notable strengths. It is the first to systematically compare mealtime assistance for cognitively impaired older adults across both hospital and nursing home settings using direct, structured observations. By moving beyond single-site or survey-based approaches, the study captured the contrasting realities of acute and long-term care, illustrating how relational, family-driven support in hospitals and technically skilled professional care in nursing homes shape feeding behaviours and nutritional outcomes. Real-time observation minimised recall bias and provided behaviourally specific insights into mechanisms contributing to inadequate intake, particularly in acute care. These findings offer an evidence base to inform training, policy development, and culturally attuned mealtime interventions.

Several limitations warrant consideration. Caregiver type was confounded with care setting—family caregivers were observed only in hospitals and professional caregivers only in nursing homes—meaning differences reflect combined effects of caregiver role and environment. The observational design lacked randomisation, and unmeasured factors such as illness severity, functional decline, or behavioural symptoms may also have influenced results. Only one mealtime episode per participant was observed; because behaviours vary across days and mealtimes, multiple observations would provide a fuller profile. The observed meal depended on the timing of the researcher’s visit, and variation related to staffing levels or workflow demands could not be assessed. Previous studies suggest staffing differs across meals, but such data were not collected here.

Measurement limitations also apply. The two observational checklists, although refined through pilot testing and supported by structured observer training, have not undergone formal psychometric validation. This may limit the precision and generalisability of behavioural comparisons. Nonetheless, reliability was strengthened through iterative pilot testing, item refinement, and research assistant training with supervised practice and calibration.

Demographic and professional information was available only for nursing home staff, not for family caregivers, limiting our ability to examine how caregiver characteristics—such as age, relationship to the patient, caregiving experience, or cultural background—may have shaped behaviours.

Given the number of statistical tests conducted, Type I error inflation due to multiple comparisons cannot be excluded. Broader sensitivity analyses (e.g., alternative modelling or stratified analyses) were not feasible in this exploratory design, and bivariate analyses with a modest sample size prevented adjustment for potential confounders. Future research should apply multivariable approaches to account for comorbidities, medication profiles, and severity of cognitive impairment.

Finally, nutrition in older adults is influenced by complex biological, psychosocial, cultural, and environmental factors, many of which fall outside the scope of this study. Despite these limitations, the study provides valuable insights into relational and technical dimensions of feeding assistance and offers a foundation for tailored training, culturally responsive mealtime strategies, and integrated models of care to improve nutritional outcomes for cognitively impaired older adults.

## 6. Conclusions

This study identified distinct yet complementary patterns of mealtime care for cognitively impaired older adults in hospital and nursing home settings. Family caregivers in hospitals demonstrated stronger relational engagement, including verbal cueing, conversation, and physical reassurance. Professional carers in nursing homes displayed greater proficiency in technical feeding skills such as mouth and lip opening techniques, swallowing support, and choking management. Despite these relational strengths, hospital patients experienced more feeding difficulties and poorer nutritional outcomes, whereas nursing home residents benefited from structured routines and consistent support from trained staff.

These findings indicate that neither caregiving model is inherently superior. Each offers unique strengths, with family caregivers providing emotional connection and cultural familiarity, and professional carers offering essential clinical and safety focused expertise. A person-centred approach that brings together both relational and technical competencies may enhance mealtime experiences and reduce malnutrition risk. Targeted training, interdisciplinary collaboration, and supportive organisational policies could help translate these combined strengths into improved nutritional outcomes.

These recommendations are based on observed behavioural patterns and do not imply causation, consistent with the exploratory nature of this study.

### Implications for Clinical Practice

This study highlights the need for mealtime care models that incorporate both relational and technical competencies. For hospitals, where family caregivers provide most feeding assistance, structured support is essential. Brief, practical training in positioning, safe feeding techniques, and recognising signs of aspiration can equip families to complement their relational strengths with essential safety skills. Clear guidance from nursing staff and accessible educational resources may further enhance the quality of care.

In nursing homes, where technical skills are already well established, staff development should place greater emphasis on relational engagement. Encouraging conversation, cultural sensitivity, and consistent person-centred communication may enhance intake and mealtime satisfaction.

At a systems level, policies should formalise family participation where appropriate and promote culturally responsive mealtime practices. Embedding these elements into staff training, care protocols, and organisational routines offers a realistic approach to improving nutritional outcomes for cognitively impaired older adults in both acute and long-term care settings.

## Data Availability

The datasets generated and/or analysed during the current study are available from the corresponding author upon reasonable request.

## Public Involvement Statement

There was no public involvement in any aspect of this research.

## Guidelines and Standards Statement

This manuscript was drafted in accordance with the Strengthening the Reporting of Observational Studies in Epidemiology (STROBE) statement for observational research.
